# Supplementary material for: Spatial distribution characteristics and influencing factors of China martial arts schools based on Baidu map API
Source: PLoS One. 2025 Mar 4;20(3):e0314588. doi: 10.1371/journal.pone.0314588 (PMC11878914; doi:10.1371/journal.pone.0314588)
Supplement: Attachment — Submitted filename: Answer questions of Reviewer2024.09.18.23.09.docx [file pone.0314588.s001.docx]

Reviewer #1:

This paper is novel as a whole. The research data and results are relatively new. The existing problem is that the research is not deep enough.
The problems in this paper are as follows:
1. “The spatial distribution of the herbaceous marsh vegetation, ecological effects of land-use change on two sides of the Hu’s Line”, these two research documents are not closely related to the research content of this paper, and it is suggested to delete them.

Answer：I have deleted the herbaceous marsh vegetation; ecological effects of land-use change on two sides of the Hu’s Line, add other closely related documents. In addition, the citation format of the references was modified.

**In part Introduction**

2. For Introduction section, the summary and analysis of the relevant research status at home and abroad are seriously inadequate. The authors should vigorously quote and expound relevant research documents and summarize and concise the technical problems existing in these research documents.

Answer：

I have re-sorted the relevant literature in the field of Wushu education and spatial distribution Baidu data in the introduction. and summarize and concise the technical problems existing in these research documents.

See introduction part

3. For "Geological Information", it should be Geographical, not Geological.

Answer：

In part 2.1, I changed “Geological Information” to “Geographical”.

4. For "Feb 2024", this month is inconsistent with the month in the abstract and should be unified and consistent.

Answer:

It should be “May 2024”, so I Make corrections in part 2.1.1

5. For the sections from 2.2.1 to 2.2.6, each section should give the number of the cited references.

Answer:

I added references number at the end of each formula.

6. The Table 1 should give the number of the cited references.

Answer：

the number of MASs in China's provinces is in Attachments (Named: Baidu map martial arts school coordinate data), the data is from Baidu Map API(<https://lbsyun.baidu.com/>), the (Table1.) is the martial arts schools’ number of provinces statistics table. So, there is no cited reference.

7. For the Figure 1, the graphic meaning of the figure is not corresponding to the content of the paper, and it is not clear, so the authors should briefly explain the figure.

Answer:

I have reinterpreted the information in Figure 1 and explained the importance of the data results in Figure 1 for this study.

8. In the section 4.1, among the influencing factors, the authors did not consider the economic factor as its obvious deficiency.

Answer:

I have added the economic influence factor (Per capita disposable income of each province) in (Table 3 and Table 4) and explained the importance of the data results. Due to the particularity of Martial Arts schools in China's institutional environment and their marginalized position in the education system, there is no obvious positive correlation between the level of economic development and Martial Arts schools in spatial distribution, but there is a certain negative correlation.

9. In the section 4.2, the number of influencing factors is small. The authors should choose more influencing factors, such as economy and culture.

Answer:

In fact, the influence of the Wushu Hometowns, the Wushu boxings numbers and the intangible cultural heritage of wushu at provincial level on the spatial distribution characteristics of Martial Arts schools represents the influence of wushu culture on Martial Arts schools. The Wushu boxings shows the popularity of regional wushu culture, and the intangible cultural heritage of wushu represents the deep degree of wushu culture in different provinces. The Wushu Hometown is a representative area where wushu culture is prevalent.

10. In the section 5, the research results mentioned above in this paper should be compared and discussed with the research results of related research literature in terms of correctness and accuracy.

Answer:

Based on the data, I will extract and analyze several key variables, including the number of provincial-level Wushu intangible cultural heritage boxing styles, the total number of Wushu boxing schools, population density, educational attainment, and per capita disposable income for each province. While the literature on Wushu hometowns has been compared and analyzed, the scope for comparison and discussion remains limited due to the scarcity of relevant research and the fragmented nature of available data.

11. In the section 6, for "socio-economic conditions", this is a very important influencing factor, and it is suggested that it should be added to the study of influencing factors in this paper.

Answer:

I chose the 2023 GDP and per capita disposable income of each province and municipality directly under the Central Government (excluding Hong Kong, Macao, and Taiwan) as the socio-economic conditions, and the overall data result was greatly affected when GDP was used as the influencing factor for analysis. Therefore, per capita disposable income was selected as the influencing factor for analysis, but the results of the two-analysis showed that the spatial distribution relationship with martial arts schools was not significant. In fact, compared with compulsory education, martial arts schools in China have higher education fees and narrow employment scope, and the development of martial arts schools in economically developed areas has not been positively affected.

Reviewer #2:

1. Although the paper provides some research background, it is better to review existing studies more comprehensively on the spatial distribution of martial arts schools and their influencing factors.

Answer:

Based on browsing more relevant research results at home and abroad, the introduction part is re-sorted out.

2. Please clarify how the sample of martial arts schools was selected and whether it is representative. Additionally, discuss the potential impact of regions not included in the study on the overall conclusions.

Answer:

we distilled this dataset to the precise names, along with the geographical coordinates—latitude and longitude-of 492 MASs, as cataloged by the end of May 2024. The latitude and longitude coordinates of the martial arts schools, after screening, can clearly reflect the number of martial arts schools at the provincial scale. It is important to note that this study deliberately excludes the regions of the Hong Kong Special Administrative Region, the Macao Special Administrative Region, and Taiwan Province from its geographical scope. The economic and educational systems in these regions differ significantly from those in mainland China, making it inappropriate to analyze the factors influencing the spatial distribution of martial arts schools on the same basis. Therefore, these regions were not included in the analysis.

3. The discussion section should more comprehensively explore the implications, limitations, and future research directions of the study. For example, consider discussing how the findings could be applied to the optimization of martial arts school layout and policy formulation.

Answer:

In the fifth part, I discussed more about the relationship between spatial distribution of Wushu schools and influencing factors, analyzed the reasons, and put forward suggestions for future development.

4. In the geographic weighted regression analysis section, could you explain the rationale for choosing these specific variables as independent variables?

Answer:

The influencing factors of the independent variables selected in the geographical weighted regression are based on many mature research results. For example, there is a significant correlation between the spatial distribution characteristics of wushu schools and the education level of the population and the spatial imbalance of the population density. When the independent variables such as economy and culture are added to the wushu hometown, the results are negatively affected. Since the number of provincial wushu intangible cultural heritage and the number of wushu townships in spatial distribution overlap to a high degree, the townships of wushu and boxing were established based on the number of wushu and boxing intangible cultural heritage. Therefore, the number of wushu and boxing intangible cultural heritage is not only consistent in quantity, culturally representative, but also closely related to wushu schools. Therefore, the hometown of martial arts and martial arts schools are positively correlated in terms of spatial distribution and cultural correlation. Due to the impact of economic factors on the overall data, adjustments must be made.
