# Supplementary material for: Spatial distribution characteristics and influencing factors of China martial arts schools based on Baidu map API
Source: PLoS One. 2025 Mar 4;20(3):e0314588. doi: 10.1371/journal.pone.0314588 (PMC11878914; doi:10.1371/journal.pone.0314588)
Supplement: Attachment — Submitted filename: Response to Reviewers.docx [file pone.0314588.s002.docx]

Specific comments

Questions:
1. “The spatial analytical endeavor unveiled a Moran's I value of 0.172, accompanied by a Z-score of 1.75 and a P-value of 0.079, signifying an uneven and clustered distribution pattern predominantly concentrated in provinces such as Shandong, Henan, Hebei, Hunan, and Sichuan.” Could you explain why a Moran's I value of 0.172 was considered significant, given that the P-value is above the conventional threshold of 0.05?

Answer:

Dear Reviewer,

Figure 1. The MASs spatial distribution type diagram

The P-value is 0.079, indicating that a core conclusion can be drawn: martial arts schools exhibit a clustered spatial distribution rather than a random one. A P-value of 0.05 is typically considered the threshold for statistical significance, with p-values less than 0.05 suggesting that the selected sample is unlikely to have occurred by chance. A P-value in the range of 0.05 to 0.10 suggests marginal significance, which in this case implies some characteristics of spatial clustering of martial arts schools, but not on a national map scale (Figure 1). Visual projections of the spatial distribution clearly show significant clustering in provinces such as Shandong, Henan, Hebei, Hunan, and Sichuan, while some martial arts schools exhibit random distribution patterns (Figure 2), consistent with the P-value of 0.079.

Moran's I = 0.172. This value indicates a modest level of spatial autocorrelation. Even though the P-value is marginal, the clustering in specific provinces (Shandong, Henan, Hebei, Hunan, and Sichuan) is practically significant. Additionally, it is worth noting that these provinces are historically significant regions where traditional Chinese martial arts have been widely practiced. The number of martial arts styles, intangible cultural heritage martial arts styles, and "Hometowns of Wushu" in these provinces provide strong statistical support for the spatial clustering of martial arts schools (see the section on Influencing Factors of CMASs). If the P-value alone cannot sufficiently explain the spatial clustering of martial arts schools, the use of ArcGIS software in this study highlights its unique value in identifying these spatial distribution patterns.

Figure 2. The distribution of martial arts schools in China

Questions:

2. In the Introduction part, “Martial Arts Schools (MASs) serve as crucial institutions for the preservation and dissemination of wushu culture, acting as the primary platform for the development of basic martial arts education in China.” The introduction mentions the marginalization of martial arts within China's compulsory education system. Could you expand on how this marginalization has evolved over the years.

Answer:

Dear Reviewer,

This is a rather complex issue, which requires a detailed and lengthy explanation within the study to clarify the underlying reasons.

First, during the Republican era of China, martial arts were honored as “Guoshu,” symbolizing the Chinese people's resistance to the derogatory label of “sick man of East Asia.” At that time, the government established the Central “Guoshu Institute” to support the development of martial arts among the public. Martial Arts were widely practiced in military academies and schools. However, many institutions, including “Guoshu institutes” and schools, were forced to close due to years of war.

Second, in the early years of the People's Republic of China (1949-1976), many martial arts organizations were either limited in development or disbanded due to national policies of the time. The need to develop competitive sports in China led to the separation of sports from the education system, forming a dedicated sports system aimed at training competitive athletes. Martial arts schools became institutions primarily focused on nurturing talent for competitive sports.

Third, during the Reform and Opening-Up period (1978-2024), China’s shift to a market economy led to the inflow of private capital into both the education and sports systems. As a result, educational institutions were categorized into public and private schools. Public institutions, managed by government departments, are formal, mainstream schools with low tuition fees. In contrast, many private schools are smaller in scale, charge higher fees, and hold a secondary status within the education system. Martial arts schools, both public and private, are categorized under the sports system. However, their curriculum is rarely aligned with higher education, and their students face low graduation rates, limited career prospects, and often lack professional teaching credentials. As a result, martial arts schools have remained marginalized within China’s education system.

The development of martial arts schools has consistently been influenced by a combination of factors, including the uneven distribution of educational resources across provinces, public attitudes toward martial arts schools, employment rates of graduates, the scale of the martial arts industry, the hierarchical status of these schools, and regional cultural factors. A comprehensive explanation of the historical evolution of martial arts schools requires extensive data and detailed analysis. Therefore, only a portion of these factors can be briefly explained in this study.

Questions:

3. Abbreviations that exist in articles should be explained when thy first appear, such as Martial Arts Schools (MASs). Please give official explanations of the abbreviations in the article.

Answer:

Dear Reviewer,

I have mentioned the full name of MASs in Abstract and introduction parts. Such as “This paper examines the spatial distribution pattern and influencing factors of Martial Arts Schools (MASs) based on Baidu map data and Geographic Information System (GIS) in China.” and “Martial Arts Schools (MASs) serve as crucial institutions for the preservation and dissemination of wushu culture, acting as the primary platform for the development of basic martial arts education in China.”

Questions:

4. In the Data and Method part, “We chose ‘martial arts school’ and ‘wushu school’ as the search terms; Leveraging the Python programming language, an extensive dataset encompassing the geographical metadata of 1,986 MASs was meticulously harvested from Baidu Maps.” How did the authors validate the accuracy of the geographical data collected via Baidu Maps API? Were there any limitations identified in the dataset that could affect the analysis?

Answer:

Dear Reviewer,

To ensure the accuracy of the statistical data regarding martial arts schools, we cross-validated the acquired data with the national list of registered martial arts schools maintained by the General Administration of Sport of China since 2016 (http://21wushu.com/ReadNews.asp?newsID=11022). As the names of martial arts schools are all Chinese names, they were translated into English in the language. Additionally, to ensure the authenticity of the sample data, we randomly selected 10% of the samples for manual verification, comparing them against official websites or confirming them via phone.

5. In “the Spatial Distribution Results Analysis of MASs” part, “The spatial distribution analysis of (MASs) reveals a Z-score of 1.75390, indicating that the probability of this spatial distribution occurring randomly is less than 10%.” How do the authors explain the geographical concentration of MASs in provinces like Shandong and Henan, and does this concentration align with historical trends in martial arts education?

Answer:

Dear Reviewer,

The selection “Wushu Hometown” and “intangible cultural heritage of wushu” puts forward strict requirements for the length of its existence. Therefore, the number of regional wushu hometowns, the number of martial arts boxing and the number of the intangible cultural heritage of martial arts can show the foundation and development trend of regional martial arts culture education. The martial arts boxings number, wushu hometowns number and wushu intangible cultural heritage of boxings number in per Province of China can strongly support this idea (see the section on Influencing Factors of CMASs).

6. In “The Selection of Influencing Factors and the Analysis of Geographical Weighted Regression Results” part. “The number of martial arts boxings, and the number of provincial wushu intangible cultural heritage of boxings have a higher degree of influence on MASs, and the coefficient of the number of martial arts boxings is as high as 0.542, which is the most important influencing factor.” How did the authors ensure that multicollinearity between variables did not affect the regression results, particularly between cultural heritage and martial arts boxings?

Answer:

Dear Reviewer,

First, in this study the Variance Inflation Factor (VIF) statistical techniques were used to assess the degree of multicollinearity among the variables. The VIF score of influencing factors are below a certain threshold (typically 10). suggest that multicollinearity is not a significant concern.

In terms of interpretation, the relationship between cultural heritage and martial arts styles is likely interlinked because regions rich in intangible cultural heritage often have a strong tradition of martial arts, which would naturally result in a higher number of martial arts styles being preserved. Thus, while these factors may seem correlated, they each contribute uniquely to explaining the distribution of MASs, with martial arts styles capturing the breadth of practice and intangible cultural heritage reflecting official recognition and preservation efforts.
